# A Representative GIIA Phospholipase A_2_ Activates Preadipocytes to Produce Inflammatory Mediators Implicated in Obesity Development

**DOI:** 10.3390/biom10121593

**Published:** 2020-11-24

**Authors:** Elbio Leiguez, Priscila Motta, Rodrigo Maia Marques, Bruno Lomonte, Suely Vilela Sampaio, Catarina Teixeira

**Affiliations:** 1 Laboratório de Farmacologia, Instituto Butantan, São Paulo 05503-900, Brazil; priscila.motta@butantan.gov.br (P.M.); rodrigo.marques@butantan.gov.br (R.M.-M.); 2 Instituto Clodomiro Picado, Facultad de Microbiología, Universidad de Costa Rica, San José 11501-2060, Costa Rica; bruno.lomonte@ucr.ac.cr; 3 Faculdade de Ciências Farmacêuticas de Riberão Preto, Universidade de São Paulo, Ribeirão Preto 14040-903, SP, Brazil; suvilela@fcfrp.usp.br

**Keywords:** phospholipase A_2_, preadipocytes, prostaglandins, adipokines, cytokines, EP receptors

## Abstract

Adipose tissue secretes proinflammatory mediators which promote systemic and adipose tissue inflammation seen in obesity. Group IIA (GIIA)-secreted phospholipase A_2_ (sPLA_2_) enzymes are found to be elevated in plasma and adipose tissue from obese patients and are active during inflammation, generating proinflammatory mediators, including prostaglandin E_2_ (PGE_2_). PGE_2_ exerts anti-lipolytic actions and increases triacylglycerol levels in adipose tissue. However, the inflammatory actions of GIIA sPLA_2_s in adipose tissue cells and mechanisms leading to increased PGE_2_ levels in these cells are unclear. This study investigates the ability of a representative GIIA sPLA_2_, MT-III, to activate proinflammatory responses in preadipocytes, focusing on the biosynthesis of prostaglandins, adipocytokines and mechanisms involved in these effects. Our results showed that MT-III induced biosynthesis of PGE_2_, PGI_2_, MCP-1, IL-6 and gene expression of leptin and adiponectin in preadipocytes. The MT-III-induced PGE_2_ biosynthesis was dependent on cytosolic PLA_2_ (cPLA_2_)-α, cyclooxygenases (COX)-1 and COX-2 pathways and regulated by a positive loop via the EP_4_ receptor. Moreover, MT-III upregulated COX-2 and microsomal prostaglandin synthase (mPGES)-1 protein expression. MCP-1 biosynthesis induced by MT-III was dependent on the EP4 receptor, while IL-6 biosynthesis was dependent on EP3 receptor engagement by PGE_2_. These data highlight preadipocytes as targets for GIIA sPLA_2_s and provide insight into the roles played by this group of sPLA_2_s in obesity.

## 1. Introduction

Obesity is a chronic low-grade inflammatory condition in which adipose tissue serves as the source of inflammatory mediators. In obesity and associated diseases, such as diabetes and cardiovascular disease, high plasma and tissue activities of secreted phospholipase A_2_ (sPLA_2_) enzymes, especially group IIA (GIIA) sPLA_2_s, have been demonstrated [1,2].

Phospholipases A_2_s (PLA_2_s) are lipolytic enzymes with important physiological functions, including cell membrane remodelling and lipid metabolism. These enzymes are classified according to their cellular localization as either intracellular PLA_2_ (iPLA_2_) enzymes with high molecular weight or sPLA_2_s, enzymes with low molecular weight. sPLA_2_s hydrolyze glycerophospholipids at the sn-2 position of the glycerol backbone, releasing fatty acids and lysophospholipids in a calcium-dependent manner. sPLA_2_s are classified into 11 groups and possess, as a common motif, a conserved His-Asp catalytic dyad. Group IIA sPLA_2_ comprises mammalian sPLA_2_s found in the inflammatory fluid of mammals and sPLA_2_s from Viperidae snake venoms. Besides their role in cell membrane physiology, mammalian group IIA sPLA_2_ are known as important autocrine and paracrine players in inflammatory processes by releasing fatty acids from cell membranes leading to production of pro-inflammatory mediators such as leukotrienes and prostaglandins [3,4,5]. Their role in metabolic diseases, such as obesity, has also been shown [1,4,6]. It is known that inhibition of sPLA_2_s, using pharmacological intervention, reduced lipid mediator’s synthesis and inflammatory parameters linked to obesity. In this sense, prostaglandin E_2_ (PGE_2_) is the most abundant lipid mediator produced by the body. This mediator is constitutively produced in all tissues by the cyclooxygenases (COX) enzymatic system and terminal PGE-synthases [7,8]. PGE_2_ is a powerful molecule carrying multiple biological effects, which are mediated by four subtypes of G protein-coupled receptors, named EP1, EP2, EP3 and EP4, depending on the tissue or cell type [7,8]. PGE_2_ is recognized as an important mediator of inflammation, pain and fever. In addition, PGE_2_ plays important roles in the regulation of proliferation and cell differentiation, and exerts anti-lipolytic actions and increases triacylglycerol levels in adipose tissue cells, contributing to lipid accumulation in these cells [9,10]. However, the molecular mechanisms triggered by GIIA sPLA_2_s that lead to the biosynthesis of PGE_2_ by adipose tissue cells are poorly known.

Preadipocytes correspond to a greater cellular fraction present in white adipose tissue and contribute significantly to the production and secretion of inflammatory mediators, such as PGE_2_ and adipokines, involved in the pathogenesis of obesity [11,12,13,14]. It has been shown that preadipocytes are target cells for a variety of inflammatory factors secreted by macrophages, which are the main cells involved in establishing an inflammatory environment in adipose tissue. In addition, when compared to mature adipocytes, preadipocytes are more responsive to inflammatory stimuli, as they offer a greater activation of the transcription nuclear factor kappa B (NF-kB) and related protein kinases [15]. Therefore, these cells may be used as a cell model for the understanding of the inductors and mechanisms involved in the development of inflammatory processes linked to obesity.

Myotoxin-III (MT-III) is a representative GIIA sPLA_2_ isolated from *Bothrops asper* snake venom that shares functional and structural similarities with mammalian pro-inflammatory sPLA_2_s of the same group [16,17,18]. MT-III is known to trigger inflammatory events in both in vivo and in vitro experimental models. Our group has previously shown that MT-III activates macrophages’ functions and induces the accumulation of lipids into these cells [19]. In addition, this enzyme is able to upregulate the differentiation of macrophages into foam cells [20], which are closely associated with diseases linked to lipid imbalance, including obesity [21,22]. On these bases, in this study, the ability of MT-III to activate proinflammatory responses in preadipocytes focusing on the biosynthesis of lipid mediators, cytokines and adipokines and the mechanisms involved in this process were investigated. In this study, we show for the first time that preadipocytes are target cells for the action of MT-III, a representative GIIA sPLA_2_, which triggers inflammatory pathways implicated in the development of obesity. The effect of MT-III involves the biosynthesis of PGE_2_, MCP-1 and IL-6 and gene expression of leptin and adiponectin. PGE_2_ biosynthesis is dependent upon the activation of cytosolic PLA_2_ (cPLA_2_)-α, COX-1, COX-2 and mPGES-1 pathways. EP3 and EP4 receptors play key roles in the release of PGE_2_ and cytokines.

## 2. Materials and Methods

### 2.1. Chemicals and Reagents

(3-(4,5-dimethylthiazol-2-yl)-2,5-diphenyltetrazolium bromide) MTT and L-glutamine were obtained from USB (Cleveland, OH, USA). Mouse mAb anti-β-actin was purchased from Sigma-Aldrich (St. Louis, MO, USA). The PGE_2_ enzyme immunoassay kit, Valeryl Salicylate, compounds NS-398, AH6809, AH23848, SC-19220, L-798106 and polyclonal antibodies against COX-1, COX-2, mPGES-1 and the EP4 receptor were purchased from Cayman Chemical Company (Ann Arbor, MI, USA). Pyrrolidine-2 (Pyr-2) was purchased from Calbiochem-Novabiochem Corp. (La Jolla, CA, USA). Secondary antibodies, anti-mouse and anti-rabbit, conjugated to HRP and nitrocellulose membrane, were obtained from GE Healthcare (Buckinghamshire, UK). The Cytometric Bead Assay (CBA) kit was purchased from BD Bioscience (San Jose, CA, USA). Gentamicin was purchased from Schering-Plough (Whitehouse Station, NJ, USA), DMSO from Amresco (Solon, OH, USA) and Dulbecco’s Modified Eagle Medium (DMEM), fetal bovine serum and real-time polymerase chain reaction (PCR) assay kit from Life Technologies (São Paulo, SP, Brazil).

### 2.2. Phospholipase A_2_ (PLA_2_)

Aspartate-49 sPLA_2_, named MT-III (Uniprot accession no.: P20474), from *B. asper* venom was purified by ion-exchange chromatography on CM Sephadex C-25 using a KCl gradient from 0 to 0.75 M at pH 7.0 as described [23], followed by RP-HPLC on a semipreparative C8 column (Vydac; 106,250 mm, 5 mm particle size), eluted at a flow rate of 2.5 mL/min with a gradient of acetonitrile (0–70%, containing 0.1% trifluoroacetic acid) over 30 min. Homogeneity was verified by SDS-PAGE, run under reducing conditions, in which a single band of 14 kDa was observed. The complete amino acid sequence of this enzyme has been described previously [23,24]. The absence of endotoxin contamination in the MT-III batches used was demonstrated by a quantitative LAL test [25], which revealed undetectable levels of endotoxin (0.125 EU/mL).

### 2.3. Cytotoxicity Assay

The cytotoxicity of MT-III and toward the 3T3-L1 preadipocyte was evaluated using the MTT assay previously described [19]. In brief, 4 × 10^3^ preadipocytes per well in DMEM, supplemented with 40 μg/mL gentamicin sulfate and 2 mM L-glutamine, were plated in 96-well plates and incubated with MT-III (0.4 μM), COX inhibitors or PGE_2_ antagonist receptors, diluted in medium or with the same volume of medium alone (control) for 1, 3, 6, 12, 24 and 48 h at 37 °C in a humidified atmosphere (5% CO_2_). MTT (5 mg/mL) was dissolved in PBS and filtered for sterilization and removal of insoluble residues. Stock MTT solution (10% in culture medium) was added to all wells in each assay, and plates were incubated for 3 h at 37 °C. Dimethyl sulfoxide (DMSO) (100 μL) was added to all wells and mixed thoroughly for 30 min, at room temperature. Absorbances were then recorded in a microtiter plate reader, at 540 nm. Results were expressed as percentages of viable cells, considering control cells incubated with medium alone as 100% viable.

### 2.4. 3T3-L1 Cell Culture and Stimulation

3T3-L1 preadipocytes obtained from the American Type Culture Collection were cultured as described [26]. Cells were processed according to the experimental protocol, in which 5 × 10^3^ preadipocytes per well were seeded in 12-wells culture plates and maintained in culture medium for 48 h before stimulation. Preadipocytes were serum-starved in DMEM with 1% (*v*/*v*) gentamicin sulfate supplemented with 1% (*v*/*v*) *L*-glutamine for 18 h prior to all treatments. Cellular homogenates were used for the Western blotting analysis of COX-1, COX-2, EP1–EP4 receptors and mPGES-1 protein expression, and supernatants of each treatment were used to measure lipid mediators PGE_2_, PGI_2_, LTB_4_ and TXA_2_ by Enzyme Immunoassay (EIA) and cytokines MCP-1, IL-6, IL-10, IL-12, TNF-α and INF-γ by CBA. Cells were stimulated with MT-III (0.4 mM) diluted in DMEM (serum free) or DMEM alone (control) for selected periods of time and maintained at 37 °C in a humidified atmosphere (5% CO_2_). To investigate the mechanism involved in the PGE_2_ and cytokine biosynthesis, selective inhibitors or antagonists were used at concentrations previously tested: 10 μM valeryl salicylate (COX-1 inhibitor) and NS-398 (COX-2 inhibitor); 10 μM SC-19220 (EP1 receptor antagonist); AH6809 (EP2 receptor antagonist) and AH23848 (EP4 receptor antagonist); and 1 μM L-798106 (EP3 receptor antagonist) [27,28,29,30,31]. All of the stock solutions were prepared in DMSO and stored at −20 °C. Aliquots were diluted in DMEM immediately before use. DMSO concentration was always lower than 1%. The viability of cells treated with inhibitors or antagonists was evaluated with MTT assay. No significant changes in cell viability were registered with any of the above agents or the vehicle at the concentrations used (data not shown).

### 2.5. Western Blotting

COX-1, COX-2, EP1–EP4 receptors and mPGES-1 protein expression from homogenate cells were detected by Western blotting. Briefly, MT-III-stimulated and non-stimulated cells were lysed with 100 mL of a sample buffer (0.5 M Tris-HCl, pH 6.8, 20% SDS, 1% glycerol, 1 M β-mercaptoethanol, 0.1% bromophenol blue) and boiled for 10 min. Samples were resolved by SDS-PAGE on 10% bis-acrylamide gels overlaid with a 5% stacking gel. Proteins were then transferred to nitrocellulose membranes using a Mini Trans-Blot (Bio-Rad Laboratories, Richmond, CA, USA). Membranes were blocked for 1 h with 5% albumin in Tris-buffered saline (20 mM Tris, 100 mM NaCl and 0.5% Tween 20, pH 7.2) and incubated overnight with primary antibodies against COX-1 and COX-2; EP1, EP2, EP3, and EP4 receptors; and mPGES-1 (1:500 dilution) or β-actin (1:3000 dilution) for 1 h at room temperature. Membranes were then washed and incubated with the appropriate secondary antibody conjugated to horseradish peroxidase. Immunoreactive bands were detected by the entry-level peroxidase substrate for enhanced chemiluminescence, according to the instructions of the manufacturer (GE Healthcare). Band densities were quantified with an ImageQuant LAS 4000 mini densitometer (GE Healthcare) using the image analysis software ImageQuant TL (GE Healthcare).

### 2.6. Eicosanoid and Cytokines Quantification

PGE_2_, PGI_2_, LTB_4_ and TXA_2_ were measured using an EIA kit, while cytokines (MCP-1, IL-6, IL-10, IL-12, TNF-α, INF-γ) were quantified using a CBA kit from supernatants of preadipocytes incubated with each treatment. Kits were used according to the instructions of the manufacturer.

### 2.7. Adipocytokines Expression by Quantitative Real-Time PCR

Quantitative polymerase chain reaction was performed as described [32]. Briefly, the total RNA from preadipocytes, incubated MT-III or DMEM alone (control) for 1, 3 and 6 h was isolated using TRIzol reagent (Invitrogen, Carlsbad, CA, USA) and reverse transcription with oligo (dT) priming was performed from 2 μg of total RNA using Superscript III (Invitrogen, Carlsbad, CA, USA). The relative expression of each transcript was determined by quantitative real-time PCR in an ABI 7000 Sequence Detection System (Applied Biosystems, Forrest City, CA, USA). Each well of the 96-well reaction plate contained a total volume of 25 µL of Power SYBR Green PCR Master Mix (Applied Biosystems). The threshold cycle (Ct) was used to determine the relative expression level of each gene by normalizing to the Ct of Glyceraldehyde-3-Phosphate Dehydrogenase (GAPDH). The method of delta–delta cycle threshold (ddCt) was used to calculate the relative fold change of each gene. Data are represented as mean + SEM.

### 2.8. Statistical Analysis

Data are expressed as mean ± SEM (*n* = 4). Multiple comparisons among groups were performed using the one-way ANOVA and, as a post-test, the Bonferroni test. Differences between experimental groups were considered significant for *p*-values < 0.05. All statistical tests were performed using Prism version 5 software (GraphPad, San Diego, CA, USA).

## 3. Results

### 3.1. MT-III Induces the Release of Lipid Mediators by Preadipocytes

**Figure 1:**
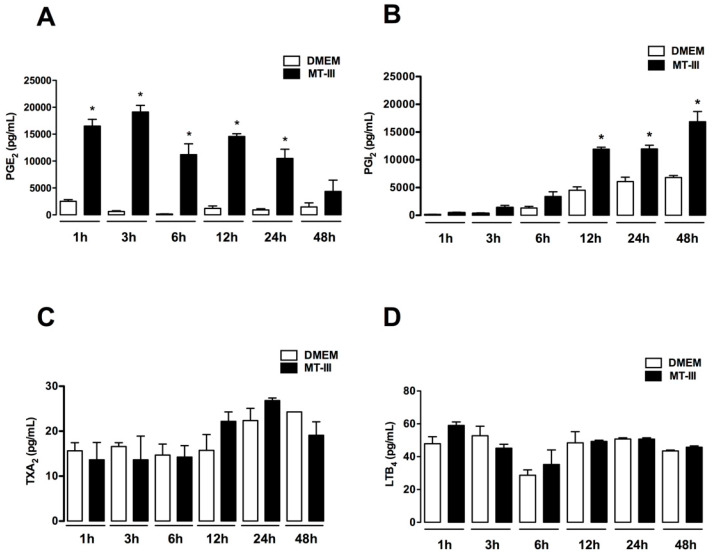
MT-III induces production of PGE_2_, PGI_2_, TXA_2_ and LTB_4_ by 3T3-L1 preadipocytes. Cells were incubated with MT-III (0.4 µM) or DMEM (control) for 1 to 48 h. Bar graphs show the MT-III-induced release of PGE_2_ (**A**), PGI_2_ (**B**), TXA_2_ (**C**) and LTB_4_ (**D**) by preadipocytes. Concentrations were quantified in culture supernatants by EIA commercial kit. Results are expressed as mean ± SEM from 3 independent experiments. * *p* < 0.05 as compared with control group (two-way ANOVA and Bonferroni posttest).

Lipid mediators are involved in lipid abnormalities and contribute to the triggering of inflammatory processes in adipose tissue [33]. Therefore, we investigated the ability of MT-III to induce the release of lipid mediators linked to inflammatory processes, such as PGE_2_, PGI_2_, TXA_2_ and LTB_4_, by cultured preadipocytes. From preliminary studies (data not shown), the submaximal concentration of 0.4 µM of MT-III was chosen for these studies as it would allow potential inhibition or exacerbation of its effects by drug treatment to be detected. As shown in Figure 1, the incubation of preadipocytes with MT-III induced a significant release of PGE_2_ (A) from 1 to 24 h and of PGI_2_ (B) from 12 to 48 h when compared with controls. However, the incubation of cells with MT-III did not alter TXA_2_ (C) or LTB_4_ (D) levels in any of the time periods evaluated. These results indicate the ability of MT-III to activate preadipocytes for the production of PGE_2_ and PGI_2_.

### 3.2. MT-III-Induced Release of PGE_2_ Is Dependent on COX-1 and COX-2 in Preadipocytes

**Figure 2:**
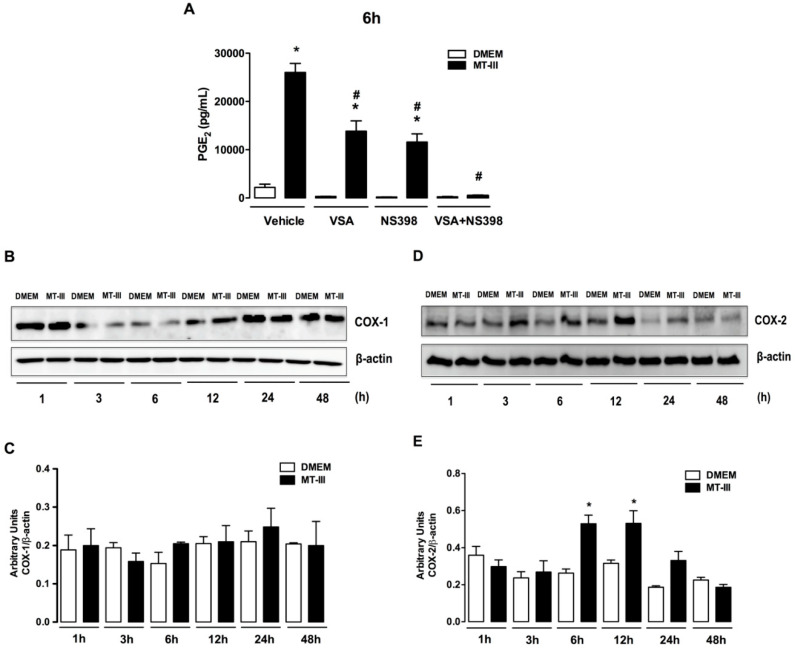
MT-III activates COX-1 and COX-2 pathways for release of PGE_2_ by 3T3-L1 preadipocytes. (**A**) Cells were incubated with either valerylsalicylate (VSA) (10 µM), or NS-398 (10 µM), or both for 1 h, followed by incubation with MT-III (0.4 µM) for 6 h. PGE_2_ concentrations were quantified in culture supernatants by EIA commercial kit. (**B**–**E**) 3T3-L1 preadipocytes were incubated with MT-III (0.4 µM) or DMEM (control) for 1 up to 48 h. (**B**) Western blotting of COX-1 and β-actin (loading control) showing immunoreactive bands. (**D**) Western blotting of COX-2 and β-actin (loading control) showing immunoreactive bands. Densitometric analysis of immunoreactive (**C**) COX-1 and (**E**) COX-2 bands. Density data (in arbitrary units) were normalized with those of β-actin. Results are expressed as mean ± SEM from 3 independent experiments. * *p* < 0.05 as compared with control group and # *p* < 0.05 as compared with MT-III group (two-way ANOVA and Bonferroni posttest).

COX-1 and COX-2 are enzymes responsible for the metabolization of arachidonic acid–generating prostanoids, such as PGE_2_ [8,34]. In order to verify the mechanism involved in the MT-III-induced biosynthesis of PGE_2_, we investigated the participation of COX-1 and COX-2 in this effect. As seen in Figure 2A, preadipocytes incubated with MT-III, in the presence of vehicle (DMSO), showed a significant release of PGE_2_ after 6 h when compared with controls. Preadipocytes treated either with COX-1 inhibitor (valerylsalicylate) or COX-2 inhibitor (NS-398) before the MT-III stimulus showed a reduction in PGE_2_ release which was statistically significant when compared to the positive control. Treatment of cells with both valerylsalicylate and NS-398 compounds abolished the MT-III-induced release of PGE_2_ when compared to the positive control. These results indicate that COX-1 and COX-2 are key enzymes involved in the release of PGE_2_, induced by MT-III, in preadipocytes. Having shown that both COX isoforms participate in the signalling pathway triggered by MT-III that leads to PGE_2_ production, we next investigated whether MT-III is able to upregulate the protein expression of COX-1 and COX-2 in preadipocytes. Our results show that preadipocytes constitutively expressed both isoforms of COX. Figure 2B,C show that COX-1 protein expression did not differ significantly between control cells and cells treated with MT-III. However, the protein expression of COX-2 was higher in cells incubated with the phospholipase A_2_ after 6 and 12 h (Figure 2D,E). Therefore, although the COX-2 isoform is constitutively expressed by preadipocytes [35], our results show that MT-III upregulates the protein expression of COX-2 but not COX-1 in preadipocytes.

### 3.3. MT-III Upregulates Protein Expression of mPGES-1 by Preadipocytes

**Figure 3:**
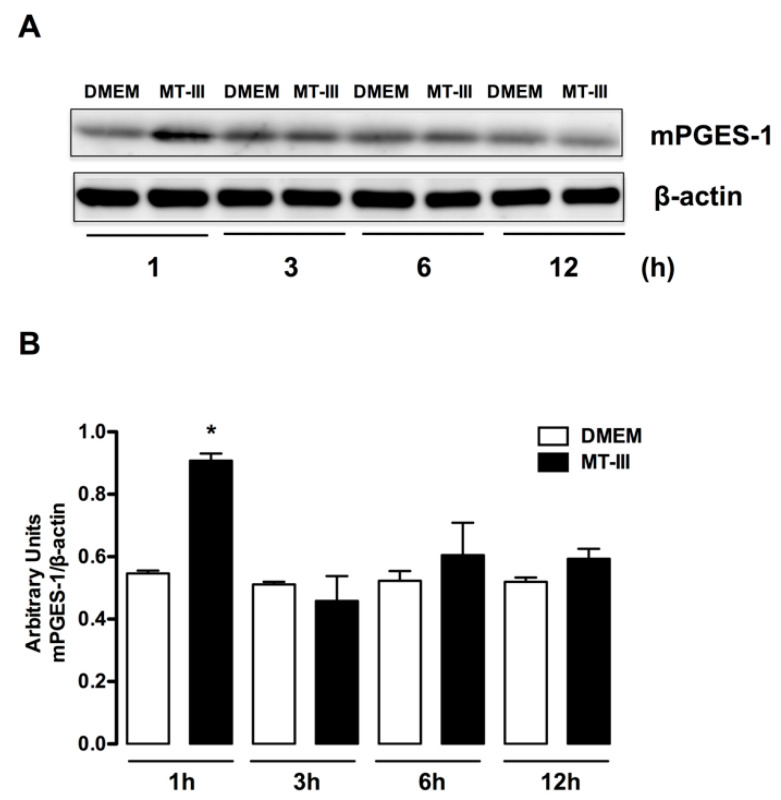
MT-III upregulates protein expression of mPGES-1 in 3T3-L1 preadipocyte. Cells were incubated with MT-III (0.4 µM) or DMEM (control) for 1 up to 12 h. (**A**) Western blotting of mPGES-1 and β-actin (loading control) showing immunoreactive bands. (**B**) Densitometric analysis of immunoreactive mPGES-1 bands. Density data (in arbitrary units) were normalized with those of β-actin. Results are expressed as mean ± SEM from 3 experiments. * *p* < 0.05 as compared with the control group (two-way ANOVA and Bonferroni posttest).

An inducible synthase responsible for the terminal synthesis of PGE_2_, mPGES-1 is upregulated in inflammatory conditions [36,37]. Based on this, we evaluated the ability of MT-III to upregulate the protein expression of this enzyme in preadipocytes. Our results show that MT-III upregulated the protein expression of mPGES-1 after 1 h of stimulation when compared with the control (Figure 3). The phospholipase A_2_ did not alter the protein expression of mPGES-1 at other time intervals evaluated. These results demonstrate that MT-III induces protein expression of mPGES-1 in preadipocytes.

### 3.4. MT-III-Induced Release of PGE_2_ Is Dependent on Cytosolic PLA_2_-α in Preadipocytes 

**Figure 4:**
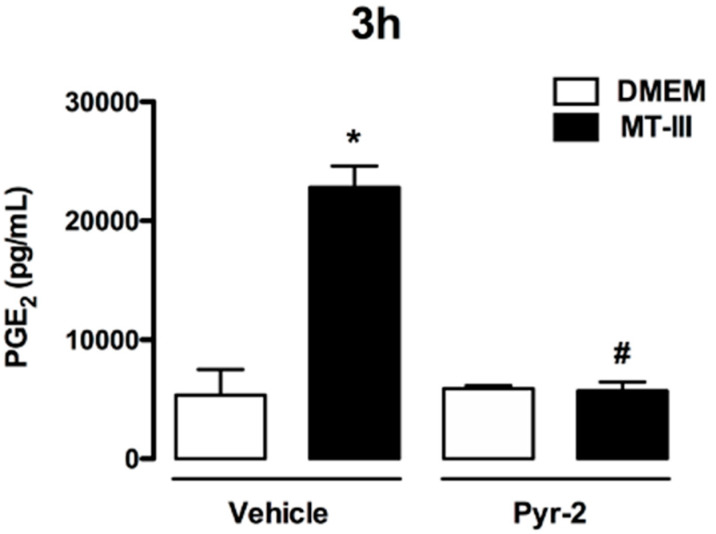
MT-III-induced PGE_2_ release is dependent on cPLA2-α in 3T3-L1 preadipocytes. Cells were incubated with Pyr-2 (1 μM) for 1 h followed by incubation with MT-III (0.4 µM) for 3 h. PGE_2_ concentrations were quantified in culture supernatants by EIA commercial kit. * *p* < 0.05 as compared with control group and # *p* < 0.05 as compared with MT-III group (two-way ANOVA and Bonferroni posttest).

It is known that sPLA_2_s cross-talk with cPLA_2_ for the synthesis of inflammatory mediators [38,39,40]. Based on this, we investigated the participation of cPLA_2_-α in the release of PGE_2_ induced by MT-III. Our results show that preadipocytes incubated with MT-III, in the presence of vehicle (positive cotrol), showed a significant release of PGE_2_ after 3 h when compared with respective control. However, pre-treatment of cells with cPLA_2_-α inhibitor (Pyr-2) abrogated the MT-III-induced release of PGE_2_ when compared to the positive control (Figure 4). These results indicate that the MT-III-induced production of PGE_2_ is dependent on cPLA_2_-α in preadipocytes.

### 3.5. MT-III-Induced Release of PGE_2_ Is Dependent on the EP4 Receptor in Preadipocytes

**Figure 5:**
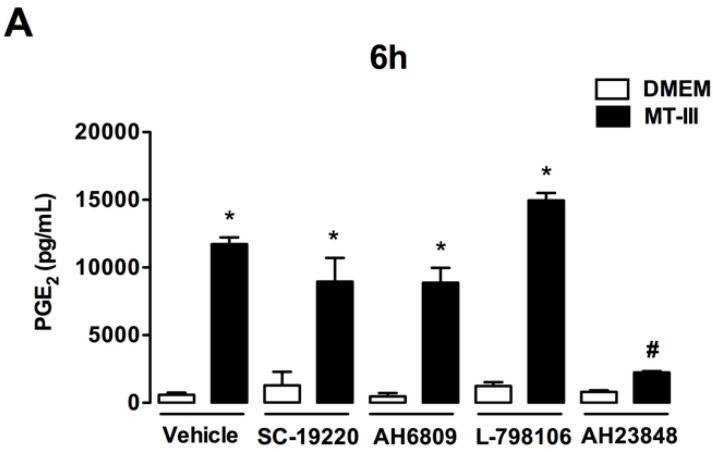

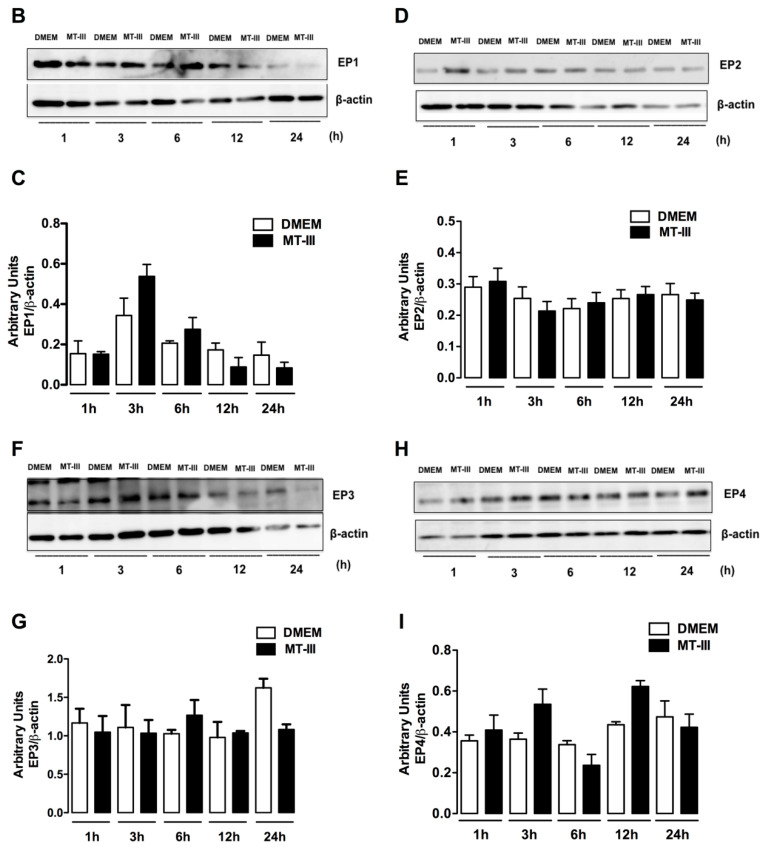
EP4 receptor participates in MT-III-induced PGE_2_ biosynthesis in 3T3-L1 preadipocytes. (**A**) Preadipocytes were incubated with SC-19220 (10 μM), AH6809 (10 μM), L-798106 (1 μM) or AH23848 (10 μM) for 1 h followed by incubation with MT-III (0.4 µM) for 6 h. PGE_2_ concentrations were quantified in culture supernatants by EIA commercial kit. (**B**–**I**) 3T3-L1 cells were incubated with MT-III (0.4 µM) or DMEM (control) for 1 up to 48 h. (**B**,**D**,**F**,**H**) Western blotting of EP1, EP2, EP3 and EP4 receptors, respectively, and β-actin (loading control), showing immunoreactive bands. (**C**,**E**,**G**,**I**) Densitometric analysis of immunoreactive bands for EP1, EP2, EP3 and EP4 receptors, respectively. Results are expressed as mean ± SEM from 3 independent experiments. * *p* < 0.05 as compared with control group and # *p* < 0.05 as compared with MT-III group (one-way ANOVA and Bonferroni posttest in (**A**) and two-way ANOVA and Bonferroni posttest in (**C**,**E**,**G**,**I**)).

PGE_2_ exerts its effects through activation of four subtypes of G protein-coupled receptors, named EP1, EP2, EP3 and EP4, and these receptors are able to regulate PGE_2_ biosynthesis [34,37,41]. It is known that the activation of the EP4 receptor by PGE_2_ may lead to the increased expression of key enzymes of biosynthesis cascade of this prostaglandin, such as COX-2 and mPGES-1 [8,41,42]. Therefore, we investigated whether PGE_2_ biosynthesis, induced by MT-III, was dependent on the activation of these receptors. As shown in Figure 5A, the incubation of preadipocytes with DMEM plus vehicle or antagonists did not cause a significant release of PGE_2_ after 6 h of incubation. Pre-treatment of cells with DMEM plus vehicle followed by incubation with MT-III (0.4 μM), for the same time period, induced a significant increase in PGE_2_ release, relative to baseline control. However, pre-treatment of cells with antagonists of EP1 (SC-19220), EP2 (AH6809) or EP3 (L-798106) receptors did not alter MT-III-induced PGE_2_ release when compared with controls. In contrast, pre-treatment of cells with the EP4 receptor antagonist (AH23848) abolished the MT-III-induced release of PGE_2_ when compared to the positive control. To better understand the involvement of the EP receptors in the effects induced by MT-III, we next analysed the protein expression of these receptors in preadipocytes stimulated with MT-III and in control cells incubated with culture medium alone. Our results show that there was no alteration in the protein expression of EP receptors in preadipocytes incubated with MT-III (0.4 μM) in any of the time periods evaluated when compared with controls (Figure 5B–I). These results indicate that PGE_2_ biosynthesis, induced by MT-III, in preadipocytes is dependent on the engagement of the EP4 receptor by PGE_2_, but not on increased protein expression of this receptor.

### 3.6. MT-III Induces Release of Inflammatory Cytokines by Preadipocytes

**Figure 6:**
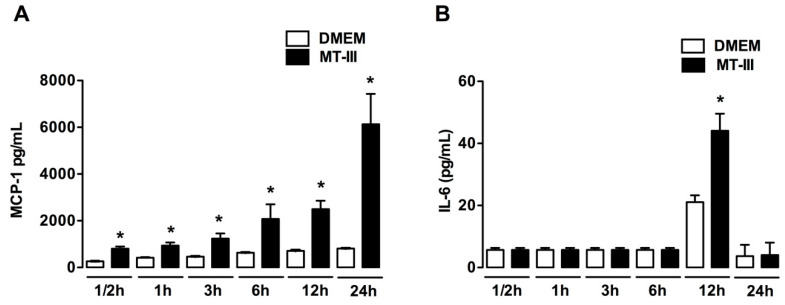
MT-III induces MCP-1 and IL-6 production by 3T3-L1 preadipocytes. Cells were incubated with MT-III (0.4 μM), or DMEM (control) for ½ up to 48 h. Bar graphs show concentrations of (**A**) MCP-1 and (**B**) IL-6 released by cells incubated with MT-III. Cytokines concentrations were quantified in culture supernatants by Cytometric Bead Array (CBA). Results are expressed as mean ± SEM from 5 experiments. * *p* < 0.05 as compared with control group (two-way ANOVA and Bonferroni posttest).

Inflammatory cytokines are found in high levels in obesity inflammatory processes and contribute to the development and maintenance of this inflammatory state [43,44,45]. On these bases, we investigated the capacity of MT-III to induce the release of the inflammatory cytokines MCP-1, IL-6, IL-10, IL-12, TNF-α and IFN-γ by preadipocytes. Figure 6A shows that MT-III induced significant release of MCP-1 from 30 min up to 24 h of incubation when compared with controls. In addition, MT-III induced significant release of IL-6 after 12 h of incubation when compared to the respective controls (Figure 6B). However, MT-III did not alter the release of IL-10, IL-12, TNF-α or IFN-γ (data not shown). In this sense, these results evidence the capacity of MT-III to induce the release of MCP-1 and IL-6 by 3T3-L1 in preadipocytes.

### 3.7. EP3 and EP4 Receptors Participate in the MT-III-Induced Release of IL-6 and MCP-1 by Preadipocytes

**Figure 7:**
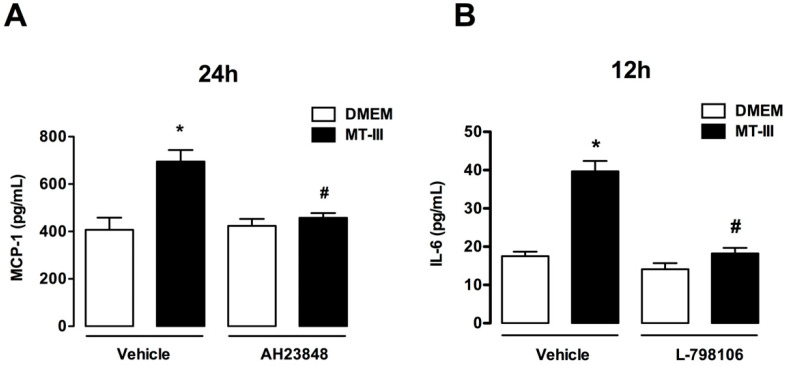
EP3 and EP4 receptors participate in the MT-III-induced release of IL-6 and MCP-1, respectively, by 3T3-L1 preadipocytes. Cells were incubated with AH23848 (10 μM) or L-798106 (1 μM) or vehicle for 1 h followed by incubation with MT-III (0,4 μM) for 12 or 24 h. Graphs show participation of EP4 receptor in the MT-III-induced release of MCP-1 (**A**) and participation of the EP3 receptor in the MT-III-induced release of IL-6 (**B**). Concentration of cytokines were quantified from culture supernatants by Cytometric Bead Array (CBA). Results are expressed as mean ± SEM from 5 experiments. * *p* < 0.05 as compared with control group and # *p* < 0.05 as compared with MT-III group (one-way ANOVA and Bonferroni posttest).

Previous studies have shown that EP3 and EP4 PGE_2_ receptors regulate the release of proinflammatory cytokines [46,47]. Therefore, we investigated the participation of EP3 and EP4 receptors in the MT-III-induced release of IL-6 and MCP-1, respectively, by preadipocytes. Figure 7A shows that the stimulation of preadipocytes with MT-III, in the presence of vehicle, significantly increased MCP-1 release after 24 h when compared with the control. Pre-treatment of preadipocytes with the EP4 antagonist (AH238481) significantly reduced the release of MCP-1 in cells stimulated with MT-III in comparison with the positive control. Similarly, pre-treatment of cells with the EP3 antagonist (L-798106) reduced the MT-III-induced release of IL-6 after 12 h, which was significant in comparison with the positive control (Figure 7B). These results indicate that EP3 and EP4 receptors participate in the release of IL-6 and MCP-1, respectively, in preadipocytes stimulated with MT-III.

### 3.8. MT-III Upregulates Gene Expression of Adipokines in Preadipocytes

**Figure 8:**
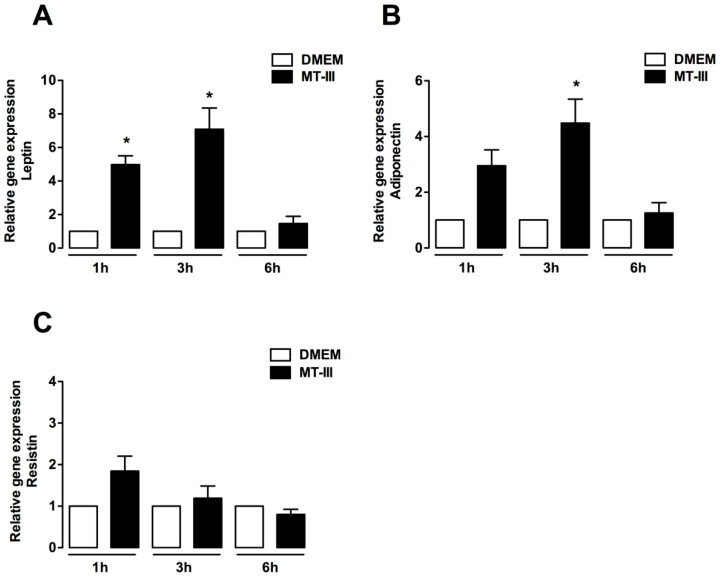
MT-III upregulates gene expression of leptin and adiponectin by 3T3-L1 preadipocytes. Cells were incubated with MT-III (0.4 μM) or DMEM (control) for 1, 3 or 6 h. Graphs show gene expression of leptin (**A**), adiponectin (**B**) and resistin (**C**) in the presence of MT-III. Concentrations of adipokines were quantified in cell lysates by qPCR. Results are expressed as mean ± SEM from 5 experiments. * *p* < 0.05 as compared with the control group (two-way ANOVA and Bonferroni posttest).

Adipokines are produced by white adipose tissue and are involved in a wide variety of physiological and pathological processes. Proinflammatory adipokines contribute to the development and maintenance of the inflammatory state in obese individuals [35,48,49]. In light of this, we investigated the ability of MT-III to induce gene expression of the adipokines leptin, resistin and adiponectin by preadipocytes. As demonstrated in Figure 8A, preadipocytes incubated with MT-III showed a significant increase in the gene expression of leptin from 1 to 3 h when compared with controls. In addition, preadipocytes incubated with MT-III showed a significant increase in the gene expression of adiponectin after 3 h (Figure 8B). However, the phospholipase A_2_ did not affect the gene expression of resistin in any of the time periods evaluated (Figure 8C). These results indicate that preadipocytes can respond to MT-III with the production of leptin and adiponectin but not resistin.

## 4. Discussion

Levels of sPLA_2_ are elevated in the serum of obese patients as well as in inflamed fat tissue [1,2,50,51]. Previous studies have implicated sPLA_2_s in metabolic diseases, including obesity [1,4,6]. However, the direct effects and mechanisms triggered by this class of enzymes on adipose tissue cells are not completely known. We herein report the ability of MT-III, a representative GIIA sPLA_2_, to activate proinflammatory pathways in preadipocytes.

Prostanoids are produced from the metabolism of arachidonic acid by the cyclooxygenases system (COX-1 and COX-2) and are implicated in events related to the development of obesity, including inflammation and the differentiation of preadipocytes into mature adipocytes [33,52]. Our results demonstrate that MT-III induced an early and sustained release of PGE_2_, followed by a late release of PGI_2_. Taking into account the marked biosynthesis of PGE_2_ in preadipocytes stimulated by MT-III and the contribution of this mediator to the inflammation process in the adipose tissue, the mechanisms involved in PGE_2_ biosynthesis, induced by MT-III, were investigated. Our findings with a pharmacological approach indicated that PGE_2_ production induced by MT-III is dependent upon the activation of COX-1 and COX-2 in preadipocytes. As an additional mechanism, MT-III upregulated COX-2 protein expression, but not COX-1 protein expression in preadipocytes. To our knowledge, this is the first demonstration that a GIIA sPLA_2_ directly activates PGE_2_ biosynthesis in preadipocytes, the precursor cells of mature adipocytes. Furthermore, our data evidence preadipocytes as target cells for GIIA sPLA_2_ action.

PGE_2_ synthases, including mPGES-1, mPGES-2 and cPGES, participate in the terminal step of PGE_2_ biosynthesis by converting PGH_2_ into PGE_2_ [53]. In contrast to mPGES-2 and cPGES, the mPGES-1 isoform is upregulated in inflammatory conditions [54,55]. During obesity, upregulation of mPGES-1 expression has been shown in the adipose tissue during adipogenic processes [56]. Accordingly, we found that MT-III increased the expression of mPGES-1 in preadipocytes. The early release of PGE_2_ correlated with mPGES-1 expression, indicating the participation of this terminal synthase in PGE_2_ biosynthetic cascade triggered by MT-III, probably via functional coupling of COX-1 and mPGES-1 in the early stage of PLA_2_ stimulation. These data reinforce the ability of GIIA sPLA_2_s to activate mechanisms in preadipocytes that contribute to the development of obesity.

It is now well recognized that mammalian GIIA sPLA_2_s do not exert their biological actions through their catalytic mechanism alone [57]. Several reports evidence that human GIIA sPLA_2_s lead to eicosanoid production by means other than by directly providing arachidonic acid (AA) through catalysis. Among the non-catalytic mechanisms described is the crosstalk between mammalian GIIA sPLA2 and cPLA2 [58,59,60,61,62]. Several lines of evidence point out that the high AA specificity of cPLA_2_-α and the lack of fatty acid selectivity in sPLA_2_s can be combined to achieve specific cellular responses [38,39,40]. In this context, our finding that inhibition of the cytosolic (cPLA_2_)-α by compound Pyr-2 abrogated the release of PGE_2_ induced by MT-III indicates that the cPLA_2_-α is a crucial partner for the effect triggered by MT-III in preadipocytes. This finding is in line with our previous data showing that MT-III increased phosphorylation of cPLA_2_-α at Ser505, a hallmark of cPLA_2_-alpha activation, in human monocytes [63]. Furthermore, although MT-III has the ability to release arachidonic acid from membrane phosphatidylcholine [63], our results evidence that the catalytic activity of MT-III does not play a role in production of PGE_2_ in preadipocytes. A similar mechanism is widely accepted for mammalian GIIA sPLA2s [57].

We further extended our knowledge of the mechanisms involved in the generation of PGE_2_ induced by MT-III by focusing on the participation of the EP4 receptor, which was shown to regulate the expression of key enzymes involved in PGE_2_ biosynthesis, including COX-2 and PGESm-1 [2,41,52,64,65,66]. Our results, showing that EP4 antagonism by compound AH23848 abolished PGE_2_ release induced by MT-III, indicate a critical role of this receptor in the effect of MT-III. These results strongly suggest that engagement of the EP4 receptor by PGE_2_ triggers a positive feedback loop regulating the biosynthetic cascade of this mediator in preadipocytes stimulated by MT-III. Activation of this positive loop likely contributes to increased levels of PGE_2_ observed throughout the period of stimulation with MT-III. In accordance with our data, studies using siRNA for knockdown EP4 gene in macrophages have shown reduced COX-2 expression upon stimuli by lipopolysaccharide [64]. In addition, our data evidenced a late release of PGI_2_, which is considered a biomarker of adipocyte differentiation [67,68], in cells stimulated by MT-III. This suggests the involvement of GIIA sPLA_2_s in the differentiation of preadipocytes. Although not investigated in this study, this hypothesis is currently being investigated in our laboratory.

Development of inflammation in the adipose tissue involves an early migration of leukocytes, mainly monocytes, into this tissue, followed by the secretion of several pro-inflammatory mediators by these cells, including cytokines, thus establishing an inflammatory environment [69,70,71,72]. Our findings showing a long-lasting release of MCP-1 in preadipocytes stimulated by MT-III strongly suggest that GIIA sPLA_2_s are implicated in the infiltration of monocytes and macrophages into adipose tissue and contribute to an inflammatory response in this tissue since MCP-1 is the key chemoattractant for monocytes during inflammatory conditions [70,72,73].

In addition, the release of IL-6 seen in preadipocytes stimulated by MT-III may contribute to the establishment of an inflammatory environment in the adipose tissue. These findings are in accordance with previous reports that levels of IL-6 are elevated in inflamed adipose tissue of obese patients that was associated with the induction of insulin resistance [74,75,76]. Furthermore, using pharmacological interference, we found that the MT-III-induced release of MCP-1 and IL-6 was dependent on EP4 or EP3 activation, respectively, in preadipocytes stimulated by MT-III. In view of previous evidence that the engagement of distinct EP receptors by PGE_2_ triggers signalling pathways linked to the biosynthesis of proinflammatory cytokines [77,78], our findings indicate that PGE_2_ biosynthesis, induced by MT-III, is an essential step for the activation of proinflammatory pathways linked to cytokine production in preadipocytes stimulated by the phospholipase A_2_.

Adipose tissue produces specific cytokines known as adipokines, which are pivotal mediators that maintain a low-grade inflammation, which characterizes obesity [79,80]. These mediators have been described as exerting autocrine and paracrine effects and regulating appetite and satiety, glucose and lipid metabolism, blood pressure regulation, inflammation and immune functions [81,82,83]. Accordingly, we found that MT-III upregulated the expression of the adipokines leptin and adiponectin in preadipocytes. Previous reports have demonstrated that leptin is able to stimulate the production of proinflammatory cytokines by macrophages and expression of adhesion molecules by endothelial cells, thus contributing to the development of the inflammatory process in the adipose tissue [84]. Therefore, this mediator may be critical for the inflammatory effects triggered by MT-III in adipose tissue by promoting key inflammatory events. Moreover, in light of the modulatory effects of adiponectin in biological systems and inflammation [85], our findings suggest that this mediator may control the inflammatory response induced by MT-III leading to a low-grade inflammation environment, which characterizes obesity. In contrast, MT-III did not affect resistin expression in preadipocytes. This may be due to the predominance of mature adipocytes over preadipocytes for the production of this mediator [86]. Although the mechanisms related to the release of adipokines by MT-III have not been presently investigated, participation of PGE_2_ and MCP-1 in the expression of leptin can be suggested since PGE_2_ and MCP-1 have been described as activators of signalling pathways leading to leptin biosynthesis [87,88]. To the best of our knowledge, this is the first demonstration that a GIIA PLA_2_ has the ability to induce the expression of adipokines in preadipocytes.

## 5. Conclusions

**Scheme 1:**
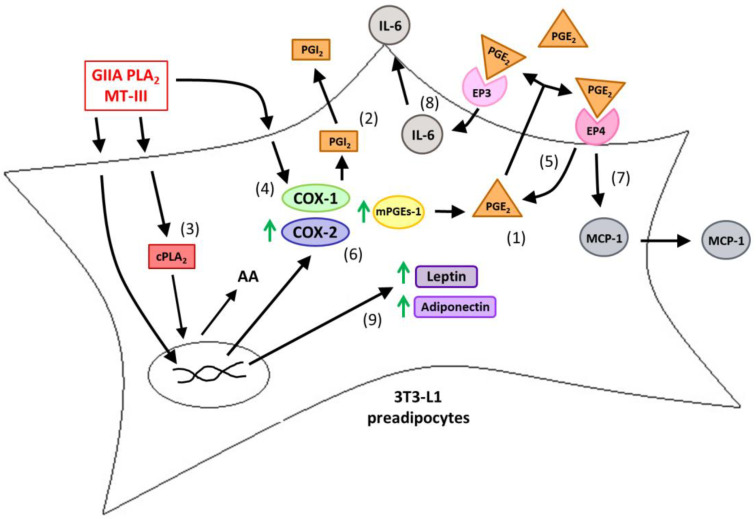
Proinflammatory pathways activated by MT-III, a GIIA snake venom sPLA_2_, in 3T3-L1 preadipocytes. (1) MT-III stimulates preadipocytes to release PGE_2_ and (2) PGI_2_; (3) PGE_2_ release induced by MT-III is dependent on cPLA_2_-α, (4) COX-1, COX-2, mPGES-1 and (5) EP4 receptor, which triggers a positive loop for PGE_2_ production; (6) MT-III up-regulates COX-2 and mPGES-1, key enzymes involved in PGE biosynthesis. Moreover, MT-III induces the release of (7) MCP-1, dependent on the EP4 receptor, and (8) IL-6, dependent on the EP3 receptor. Furthermore, (9) MT-III up-regulates leptin and adiponectin gene expression

In this study, we demonstrate for the first time the ability of a representative GIIA phospholipase A_2_, MT-III, to directly activate preadipocytes to release PGE_2_ and the critical role of this mediator, acting via receptors EP3 and EP4, in inflammatory responses induced by this sPLA_2_. MT-III also induced release of PGI_2_, MCP-1 and IL-6 but not TNF-α, INF-γ, IL-12 or IL-10, and upregulated the expression of leptin and adiponectin. The MT-III-induced PGE_2_ biosynthesis was dependent on the activation of cPLA_2_-α, COX-1 and COX-2 pathways and positively regulated by the EP4 receptor. As an additional mechanism, MT-III upregulated COX-2 and mPGES-1 protein expression. MCP-1 biosynthesis induced by this sPLA_2_ was dependent on the activation of the EP4 receptor, while IL-6 biosynthesis was dependent on the EP3 receptor in preadipocytes. Taken together, these findings provide evidence of a new target cell of the action of GIIA sPLA_2_s, extending the knowledge of the effect of this class of enzymes in the adipose tissue (Scheme 1) given new insights into the roles of GIIA sPLA_2_s in obesity and associated disorders.
